# Endocrine and uterine hemodynamic differences between intra- and interspecific equine pregnancies during early gestation

**DOI:** 10.1007/s11259-026-11472-1

**Published:** 2026-08-25

**Authors:** Diego Guedes Campos, Isabela Syllos Campos, Paulo Henrique Hümmelgen Silva, Beatriz Bringel, Robert H. Douglas, Aline Emerim Pinna, Julio Cesar Ferraz Jacob, Valério Marques Portela, Carolina dos Santos Amaral, Alfredo Quites Antoniazzi

**Affiliations:** 1 Laboratory of Reproductive Biotechnology (BIOREP), Federal University of Santa Maria (UFSM), Santa Maria, RS Brazil; 2 Department of Veterinary Pathology and Diagnostic Imaging, Fluminense Federal University (UFF), Niterói, RJ Brazil; 3 BET Reproductive Laboratory, Lexington, KY USA; 4 Department of Animal Reproduction and Evaluation, Institute of Animal Science (IZ), Federal Rural University of Rio de Janeiro (UFRRJ), Seropédica, RJ Brazil

**Keywords:** Equine pregnancy, Interspecific reproduction, Conceptus origin, Progesterone, ECG, Uterine hemodynamics

## Abstract

Interspecific equine pregnancies (mare x donkey) are associated with altered endocrine patterns and increased pregnancy loss; however, the relationship between hormonal dynamics, luteal activity, and uterine hemodynamics in relation to conceptus origin remains incompletely understood. This study aimed to evaluate the influence of conceptus origin on endocrine profiles, supplementary corpora lutea (SCL) formation, and uterine hemodynamics, and to investigate the relationships among these variables. Twenty mares were initially included; however, six were excluded due to pregnancy loss or incomplete follow-up, resulting in a final sample of 14 mares (six stallion-derived and eight donkey-derived pregnancies). Serum progesterone (P4), equine chorionic gonadotropin (eCG), SCL number, and uterine artery Doppler indices, including pulsatility index (PI) and resistivity index (RI), were evaluated at Days 49, 63, 84, and 98 of gestation. Donkey-derived pregnancies showed significantly lower P4 and eCG concentrations, reduced SCL formation, and lower uterine PI and RI compared to stallion-derived pregnancies (*p* < 0.05). While stallion-derived pregnancies remained relatively stable over time, donkey-derived pregnancies showed progressive declines in P4 and eCG concentrations, accompanied by reductions in uterine PI and RI. Positive correlations were observed between P4 and PI (*r* = 0.846; *p* < 0.001) and between eCG and PI (*r* = 0.733; *p* = 0.015). All pregnancies were maintained to term. These findings indicate that conceptus origin is associated with distinct endocrine and uterine hemodynamic patterns, supporting integrated hormonal–vascular adaptations in equine pregnancy.

**Supplementary Information:**

The online version contains supplementary material available at https://doi.org/10.1007/s11259-026-11472-1.

## Introduction

The maintenance of pregnancy in mares (*Equus caballus*) depends on the coordinated interaction of endocrine, vascular, and immunological mechanisms that support conceptus development. In this context, progesterone (P4) plays a central role in maintaining uterine quiescence and endometrial receptivity (Conley 2016; Meira et al. 2018; Campos et al. 2023). In mares, reproductive success depends on continuous P4 production, and a positive correlation has been demonstrated between circulating P4 concentrations and pregnancy rates (Boeta and Zarco 2005).During early gestation, the formation of endometrial cups promotes the secretion of equine chorionic gonadotropin (eCG), which stimulates the development of supplementary corpora lutea (SCL) and reinforces luteal progesterone production until the establishment of placental steroidogenesis, a transition known as the luteo-placental shift (Allen and Moor 1992; Allen and Stewart 2001; Brinsko et al. 2011; Boeta and Zarco 2012; Murphy 2018; Teixeira et al. 2020; Campos et al. 2023).

The eCG secreted by the endometrial cups, which form between days 36 and 38 of gestation through the invasion of chorionic girdle trophoblastic cells into the endometrium, induces SCL development via binding to luteinizing hormone (LH) receptors (Allen and Stewart 2001; Conley 2016). This process occurs because mares exhibit ovarian follicular waves stimulated by periodic increases in follicle-stimulating hormone (FSH), occurring every 10 to 12 days and persisting during the first months of pregnancy. While elevated P4 concentrations suppress ovulation of dominant follicles until approximately day 30, the LH-like activity of eCG can stimulate ovulation after day 35, resulting in SCL formation through luteinization of anovulatory follicles and ovulation of subordinate follicles (Urias-Castro et al. 2017). From the twelfth week of gestation (Days 77–84), P4 secretion progressively declines as the corpora lutea and endometrial cups regress, whereas placental production of 5α-dihydroprogesterone progressively increases and exceeds circulating P4 concentrations around Weeks 15–16 (Days 105–112), thereby supporting pregnancy until term.

In contrast, interspecific pregnancies between mares and donkeys (*Equus asinus*) present important physiological challenges. Reduced eCG production, lower circulating P4 concentrations, and decreased SCL formation have been consistently reported and are associated with an increased risk of pregnancy loss (Allen et al. 1985; Allen and Short 1997; Boeta and Zarco 2005; Urias-Castro et al. 2017; Canisso et al. 2019; Carluccio et al. 2020; Fanelli et al. 2023). Despite these advances, the etiology of embryonic and fetal loss in interspecific equine pregnancies remains incompletely understood, highlighting the need for further investigation, particularly in the context of mule production, which is a widespread practice in Brazil (Campos et al. 2023). Importantly, these endocrine differences do not result from a direct effect of the sire, but rather from the genotype of the conceptus and its interaction with the maternal endometrium, particularly through trophoblastic signaling and endometrial cup function (Allen and Stewart 2001; Boeta and Zarco 2005, 2012).

In parallel, uterine vascular adaptation represents a critical component of pregnancy maintenance Doppler ultrasonography enables non-invasive, real-time assessment of hemodynamic parameters and has been established as a fundamental tool in equine reproductive research (Ginther and Utt 2004; Ginther 2007, 2014; Ferreira and Meira 2011). Spectral Doppler provides quantitative indices such as the pulsatility index (PI) and resistivity index (RI), which are widely used as indirect estimates of vascular resistance and tissue perfusion (Bollwein et al. 2004, 2016; Silva et al. 2006). In pregnant mares, uterine hemodynamics reflect maternal vascular adaptation and directly contributes to conceptus support (Bollwein et al. 2004; Ousey et al. 2012). Previous studies have characterized uterine artery Doppler indices in mares carrying stallion- or donkey-derived pregnancies (Lemos et al. 2017; Oliveira et al. 2026), demonstrating that conceptus origin influences uterine hemodynamic adaptation and that Doppler indices reported for intraspecific pregnancies cannot be directly extrapolated to interspecific pregnancies.

Although endocrine alterations associated with interspecific equine pregnancies have been extensively described, comparatively little information is available regarding uterine hemodynamic adaptations according to conceptus origin. Furthermore, studies integrating endocrine, luteal, and uterine vascular parameters within the same experimental framework are scarce. Previous studies have independently characterized endocrine alterations, including reduced eCG secretion, lower circulating progesterone concentrations, and decreased supplementary corpus luteum formation (Boeta and Zarco 2005; Urias-Castro et al. 2017), as well as uterine hemodynamic patterns assessed by Doppler ultrasonography (Lemos et al. 2017; Oliveira et al. 2026). However, the relationship between endocrine function, supplementary luteal activity, and uterine vascular adaptation has not been investigated within the same experimental framework. Understanding these interactions may provide new insights into the physiological mechanisms that support pregnancy maintenance in mares carrying conceptuses of different paternal origin.

Therefore, the present study aimed to comparatively evaluate endocrine parameters (progesterone [P4] and equine chorionic gonadotropin [eCG]), luteal activity (supplementary corpus luteum [SCL] formation), and uterine hemodynamic indices (pulsatility index [PI] and resistivity index [RI]) in stallion- and donkey-derived pregnancies, as well as to investigate the associations among these variables during early equine gestation.

## Materials and methods

### Experimental design and ethical approval

This study was designed as a prospective longitudinal cohort and conducted during the physiological breeding season at the experimental farm of the Instituto Vital Brazil, located in Cachoeiras de Macacu, Rio de Janeiro, Brazil (22°27′45″ S, 42°39′11″ W; altitude: 57 m). All experimental procedures were approved by the Animal Ethics Committee of the Universidade Federal Fluminense (CEUA/UFF; protocol no. 001/19).

### Animals and management conditions

A total of 20 Mangalarga Marchador mares, clinically healthy and aged between 5 and 15 years, which established pregnancy following artificial insemination, were initially enrolled in the study. Prior to inclusion, all mares underwent a complete reproductive evaluation, including transrectal ultrasonography, endometrial cytology, low-volume uterine lavage for microbiological culture, and vaginoscopy. Only mares with normal reproductive parameters were included, defined as endometrial cytology showing < 2% neutrophils in smears prepared from low-volume uterine lavage samples and evaluated under 400× magnification, together with absence of bacterial growth in microbiological cultures of uterine lavage fluid.

During the follow-up period, six mares were excluded before the first experimental time point (Day 49 of gestation). Four mares (20.0%) experienced pregnancy loss between approximately Days 30 and 50 of gestation, including one of seven stallion-derived pregnancies (14.3%) and three of eleven donkey-derived pregnancies (27.3%), whereas two mares (10.0%) were excluded because of incomplete follow-up.

Thus, 14 mares were included in the final analysis, comprising six stallion-derived and eight donkey-derived pregnancies. None of the exclusions were associated with experimental procedures. Considering the longitudinal design, only mares that maintained a viable pregnancy up to the beginning of the experimental evaluations and completed all time points were included in the analysis, ensuring consistency for repeated measures comparisons.

Mares were maintained under a semi-intensive management system in paddocks with elephant grass (Pennisetum purpureum), and supplemented with commercial concentrate feed, wheat bran, and mineral mixture, with free access to water. Body condition score (BCS) was assessed using a 1–9 scale (Henneke et al. 1983), and only mares with scores between 4 and 6 were included in order to minimize potential nutritional influences on endocrine parameters.

### Reproductive procedures and experimental grouping

Follicular development was monitored by transrectal palpation and ultrasonography. Ovulation was induced by intramuscular administration of 250 µg of deslorelin acetate (Strelin^®^, Botupharma, Brazil) when a dominant follicle reached a diameter ≥ 35 mm.

Artificial inseminations were performed using doses containing 1 × 10⁹ progressively motile spermatozoa diluted in a commercial skim milk–based extender (BotuSemen^®^, Botupharma, Brazil), which was used for both stallion and donkey semen. Semen was obtained from one stallion and two donkeys, all of proven fertility, and the same insemination protocol was applied to all mares to ensure standardization between groups. According to the breeding management adopted at the farm, mares underwent a maximum of two artificial insemination cycles. Among the mares that established pregnancy and were enrolled in the study, 12 of 20 pregnancies (60.0%) were achieved after the first insemination cycle and eight (40.0%) after the second cycle. Specifically, five of eight stallion-derived pregnancies (62.5%) and seven of 12 donkey-derived pregnancies (58.3%) were established after the first insemination cycle, whereas three (37.5%) and five (41.7%), respectively, were established after the second cycle.

Mares were classified according to conceptus origin, defined by the species of the sire used for insemination, and were retrospectively grouped into stallion-derived (*n* = 6) and donkey-derived (*n* = 8) pregnancies. All inseminations were performed during the physiological breeding season (November to March).

Group allocation was not randomized, as the assignment of mares to each sire species followed a pre-established breeding program defined by the farm owner, based on zootechnical objectives including the production of offspring with specific genetic characteristics for breed exhibitions and competitions. To assess baseline comparability, mare age and body condition score were compared between groups prior to the experimental period, and no significant differences were identified.

### Pregnancy diagnosis and experimental timeline

Pregnancy was diagnosed on Day 12 post-ovulation by transrectal B-mode ultrasonography, based on visualization of the embryonic vesicle. Ovulation (Day 0) was defined as the disappearance of the dominant follicle followed by the subsequent formation of a corpus luteum.

Following pregnancy confirmation, mares were monitored weekly to assess gestational development and ovarian activity. Pregnancy loss was defined as the absence of a viable embryo or fetus in consecutive ultrasonographic examinations.

Four standardized time points were established for data collection (Days 49, 63, 84, and 98 of gestation). These time points were selected to represent the period of maximal endometrial cup activity and equine chorionic gonadotropin (eCG) secretion, which is associated with luteotrophic support and the formation of supplementary corpora lutea (Urias-Castro et al. 2017; Vilanova et al. 2019). Accordingly, evaluations were initiated on Day 49 of gestation, when eCG secretion and its physiological effects are well established. The present study was therefore specifically designed to characterize endocrine, luteal, and hemodynamic patterns during this physiologically relevant gestational window rather than as a comprehensive evaluation of early equine pregnancy as a whole.

### Ultrasonographic and Doppler evaluation

**Fig. 1:**
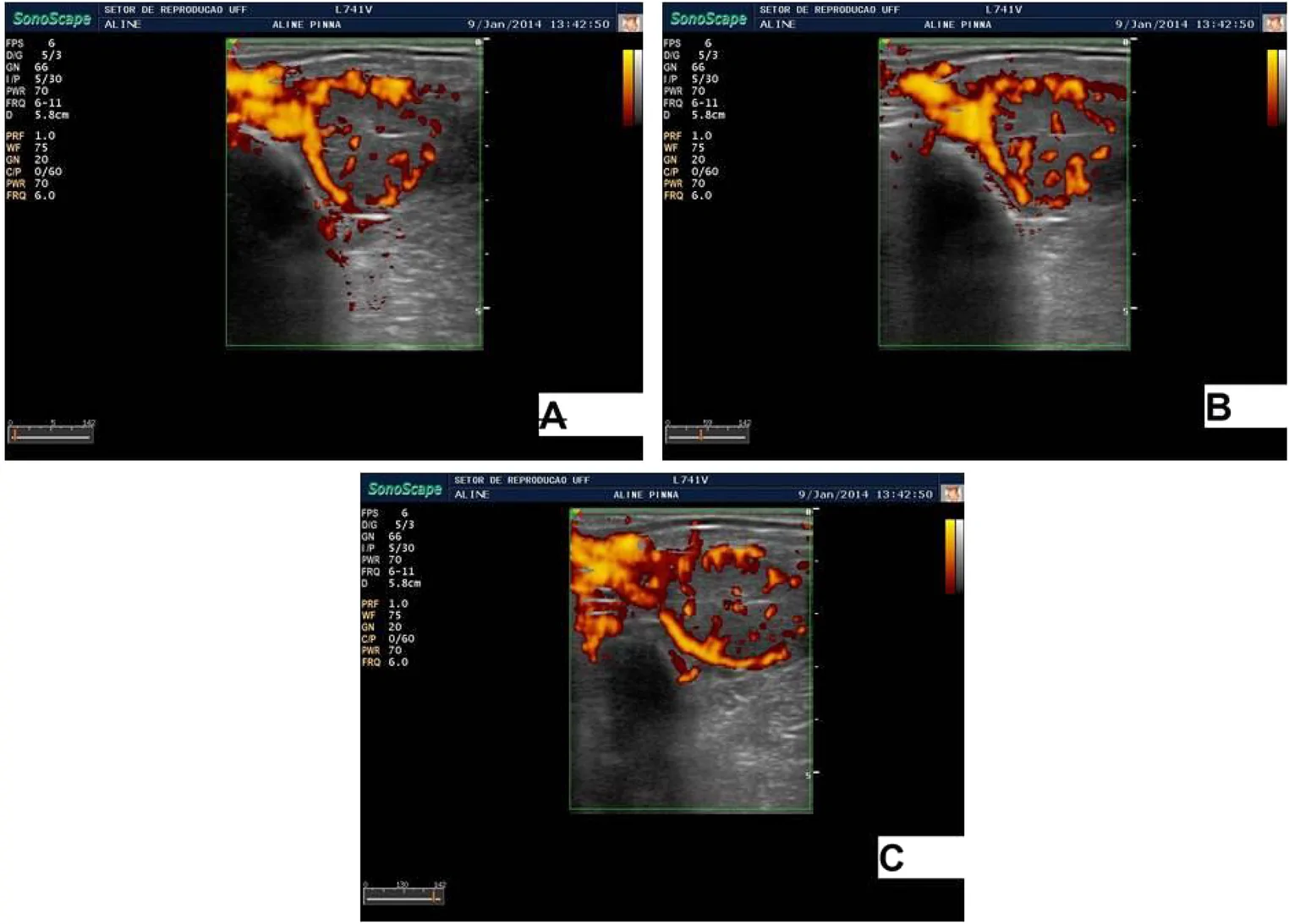
Capture of the most representative corpus luteum images obtained by power Doppler ultrasonography. (**A**, **B**, **C**) Frozen frames from a video representing the largest luteal areas containing the greatest amount of color signals

Ultrasonographic and Doppler evaluations were performed using a SonoScape S2V6 system (Shenzhen, China) equipped with a 3–7 MHz transrectal transducer, following a standardized protocol previously established by our research group (Ferreira et al. 2020; Oliveira et al. 2026). Luteal vascularization was assessed using power Doppler with standardized settings (gain: 120; frequency: 5 MHz; depth: 8.3 cm; power: 70%). Luteal perfusion was first evaluated subjectively in real time by two experienced operators, who independently estimated the proportion of the luteal cross-sectional area presenting color Doppler signal, expressed as a percentage ranging from 0% to 100%. The mean value between operators was used for analysis; in cases of discrepancy greater than 15% points, the examination was repeated and consensus was reached by direct discussion. Doppler examinations were recorded as video files, from which three representative images per corpus luteum were selected, corresponding to the largest luteal area with the highest color signal, excluding the vascular pedicle region (Fig. 1).

Selected images were exported in TIFF format, processed in Adobe Photoshop for removal of unwanted color signals, and then subjected to histogram analysis using ImageJ, in which colored pixels were quantified by applying a 256-level grayscale conversion (Ferreira et al. 2020). Spectral Doppler measurements of PI and RI were obtained independently for the uterine artery branch supplying the pregnant uterine horn and for the branch supplying the non-pregnant uterine horn, allowing bilateral hemodynamic assessment at each time point.

Supplementary corpora lutea (SCL) were defined as luteal structures formed after ovulatory events additional to the primary corpus luteum, detected during weekly ultrasonographic monitoring performed throughout the entire experimental period from Day 12 post-ovulation onward. At each weekly examination, both ovaries were systematically evaluated in multiple planes, and all identifiable follicles and luteal structures were individually measured, characterized, and recorded in individual mare tracking spreadsheets, allowing continuous longitudinal topographic mapping of all ovarian structures across consecutive examinations.

Identification of SCL was based on the combined application of B-mode and power Doppler ultrasonography at each weekly examination. In B-mode, the primary criterion was ultrasonographic evidence of follicular collapse, defined as the disappearance of a follicle with a diameter ≥ 35 mm followed by the formation of a collapsed, hypoechoic to heterogeneous structure at the subsequent weekly examination.

Power Doppler was systematically applied to both ovaries at each examination. True SCL were characterized by peripheral and progressively central vascularization consistent with functionally active luteal tissue, whereas structures lacking this vascular pattern were not classified as SCL. Follicles that failed to rupture and progressively developed increased intrafollicular echogenicity, follicular wall thickening, and absence of collapse were classified as hemorrhagic anovulatory follicles (HAF) and excluded from the SCL count, following previously established ultrasonographic criteria (Cuervo-Arango and Domingo-Ortiz 2011). The slower luteinization kinetics of HAF relative to true corpus luteum development (Cuervo-Arango and Domingo-Ortiz 2011) further reduces the likelihood of misclassification within weekly examination intervals. This longitudinal mapping approach ensured that ovarian structures could be reliably distinguished across consecutive examinations, preventing duplicate counting.

**Fig. 2:**
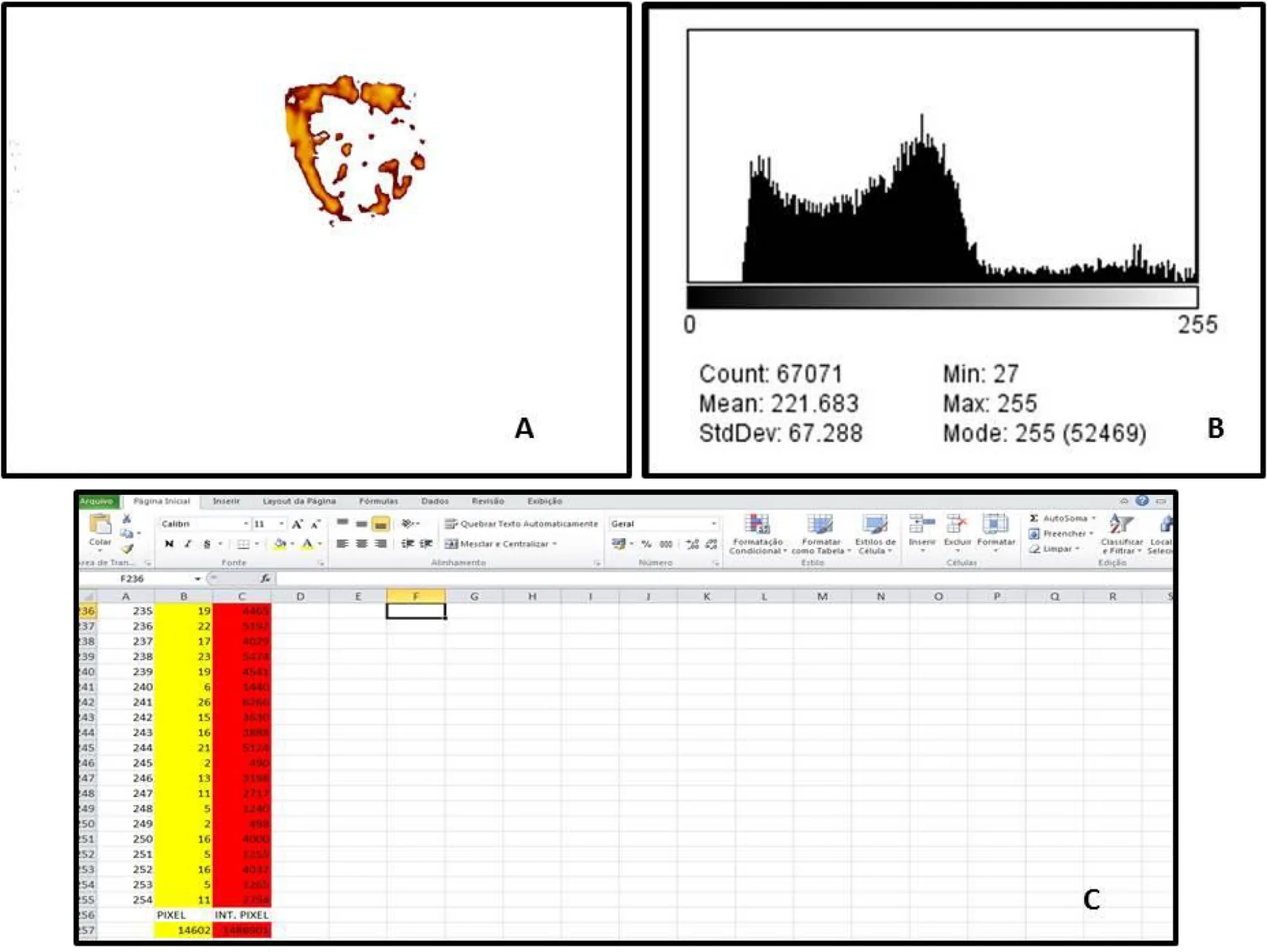
Representative corpus luteum images obtained by power Doppler ultrasonography. (**A**) TIFF-format image modified in Adobe Photoshop to remove unwanted color signals; (**B**) Histogram analysis performed using ImageJ software to calculate the number of pixels in the image; (**C**) Pixel and intensity values organized in Excel

In addition, luteal perfusion was objectively evaluated by quantifying the area of colored pixels (PIX). Selected images were exported in TIFF format and processed for quantitative analysis using ImageJ software (National Institutes of Health, USA), as illustrated in Fig. 2.

**Fig. 3:**
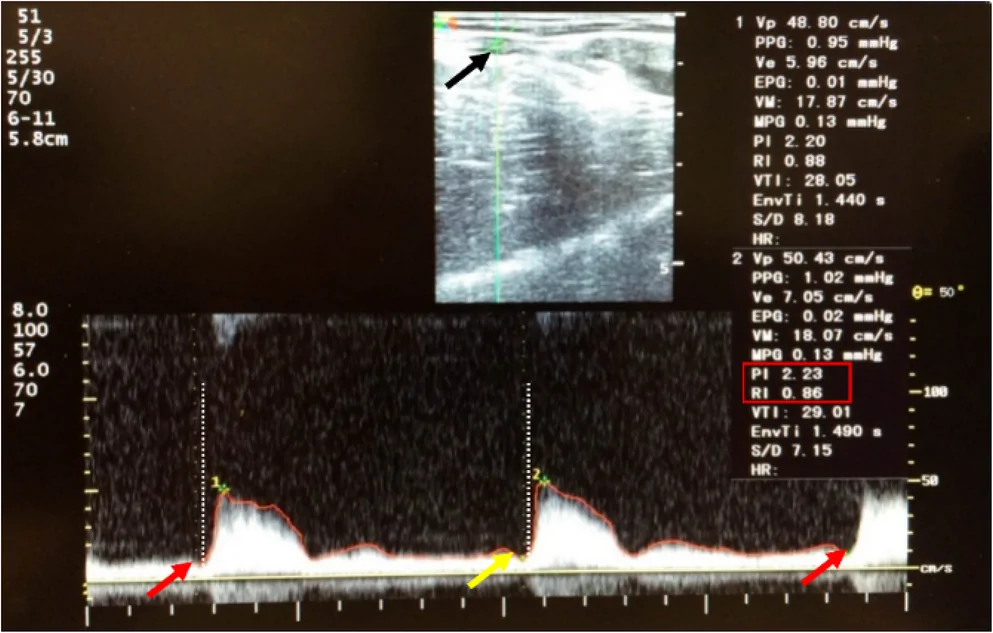
Spectral Doppler ultrasonography of the dorsal branches of the uterine arteries for evaluation of the resistance index (RI) and pulsatility index (PI). The red box identifies the Doppler sample volume used to generate the spectral waveform for calculation of RI and PI. The black arrow indicates the position of the ultrasound cursor on the uterine artery. The onset of systole and the end of diastole are indicated by the red and yellow arrows, respectively. Source: personal archive

Uterine hemodynamics were evaluated by spectral Doppler. Pulsatility index (PI) and resistivity index (RI) were measured in uterine arteries, specifically in branches originating from the external iliac artery near its bifurcation (Fig. 3). Measurements were obtained over three consecutive cardiac cycles, and mean values were calculated. Acquisition parameters were standardized (gain: 90; frequency: 7 MHz; pulse repetition frequency: 4.5 kHz; depth: 8.3 cm; power: 70%). Both pregnant and non-pregnant uterine horns were evaluated.

### Hormonal analyses

Blood samples were collected by jugular venipuncture into vacuum tubes without anticoagulant. Samples were allowed to clot at room temperature and subsequently centrifuged at 800 × g for 15 min. The serum was separated, aliquoted into microcentrifuge tubes, and stored at − 20 °C until analysis.

Serum progesterone (P4) concentrations were determined by solid-phase radioimmunoassay (RIA) using a commercial kit (RIA Progesterone, Beckman Coulter IM1188; Immunotech s.r.o., Prague, Czech Republic) previously used in equine studies (Barbosa et al. 2025), following the manufacturer’s instructions. The assay had a limit of detection of 0.04 ng/mL and a measurement range of 0.04 to 50 ng/mL, with intra- and inter-assay coefficients of variation of 4.1% and 6.3%, respectively, as determined in our laboratory. According to the manufacturer’s package insert (IFU-IM1188-04), the main cross-reacting steroids were 5α-pregnanedione (14.11%), 5β-pregnanedione (6.26%), 20α-dihydroprogesterone (0.84%), and 17α-hydroxyprogesterone (0.66%); cross-reactivity with cortisol was below the limit of detection.

Equine chorionic gonadotropin (eCG) concentrations were measured using a sandwich enzyme-linked immunosorbent assay (ELISA) (PMSG ELISA, DRG Instruments GmbH, EIA-1298, Marburg, Germany) previously used in equine studies (Urias-Castro et al. 2017), according to the manufacturer’s instructions. The assay sensitivity was 3 IU/mL. Intra-assay coefficients of variation were 7.1% for low values and 11.6% for high values, and the inter-assay coefficient of variation was 10%. Cross-reactivity with equine FSH and LH was present at low levels but eliminated by performing sample dilutions of 1:100 with Zero Standard prior to analysis, as recommended by the manufacturer. No cross-reactivity was observed with human chorionic gonadotropin (hCG). Only samples with values above the assay detection limit were included in quantitative analyses, and undetectable values were excluded to prevent analytical bias.

### Statistical analysis

Statistical analyses were performed using SPSS software (version 26.0; IBM Corp., Armonk, NY, USA). Data normality was assessed using the Shapiro–Wilk test.

Longitudinal variables, including progesterone (P4), equine chorionic gonadotropin (eCG), luteal vascularization (subjective and objective), pulsatility index (PI), and resistivity index (RI), were analyzed using two-way repeated measures analysis of variance (ANOVA), with group (conceptus origin), time (days of gestation), and their interaction (group × time) as fixed effects. When significant effects were detected, post hoc multiple comparisons were performed using the Sidak adjustment.

Associations between endocrine and hemodynamic variables were assessed using Spearman’s rank correlation coefficient and were considered exploratory due to the limited sample size.

Statistical significance was set at *p* < 0.05.

## Results

### Study population and baseline characteristics

A total of 20 mares were initially enrolled in the study. Six mares were excluded prior to Day 49 of gestation due to pregnancy loss (*n* = 4) or incomplete follow-up (*n* = 2). The final analysis included 14 mares that maintained pregnancy to term, comprising six stallion-derived and eight donkey-derived pregnancies.

At the first evaluation time point (Day 49 of gestation), mares carrying stallion-derived pregnancies exhibited higher serum progesterone (P4) and equine chorionic gonadotropin (eCG) concentrations, a greater number of supplementary corpora lutea (SCL), and higher uterine artery Doppler indices (pulsatility index [PI] and resistivity index [RI]) in both pregnant and non-pregnant uterine horns compared with mares carrying donkey-derived pregnancies (*p* < 0.05; Table 1).

**Table 1:** Endocrine, luteal, and uterine hemodynamic variables in mares carrying stallion- and donkey-derived pregnancies at Day 49 of gestation

Parameter	*n*	Stallion			*n*	Donkey			*P*-value
Day 49 of gestation									
P4 (ng/mL)	6	25.6	±	12.2	8	14.3	±	5.1	0.035
eCG (IU/mL)	6	88.7	±	7.8	4	56.0	±	9.8	0.0003
Subjective evaluation (score)	6	16.7	±	11.7	4	18.8	±	8.5	0.77
Objective evaluation (PIX)	6	5015	±	3091	4	2890	±	294	0.15
PI (pregnant uterine horn)	6	2.16	±	0.18	8	1.35	±	0.08	< 0.0001
PI (non-pregnant uterine horn)	6	1.93	±	0.09	8	1.17	±	0.09	< 0.0001
RI (pregnant uterine horn)	6	0.82	±	0.04	8	0.65	±	0.09	0.0004
RI (non-pregnant uterine horn)	6	0.79	±	0.08	8	0.42	±	0.13	< 0.0001

Additionally, four mares in the donkey-derived group did not develop detectable supplementary corpora lutea throughout the experimental period and exhibited persistently undetectable eCG concentrations. Given the absence of eCG stimulation, these mares also did not develop accessory luteal structures. Therefore, they were excluded from quantitative analyses involving eCG, as well as from analyses related to supplementary corpus luteum formation and luteal vascularization, and were considered only for descriptive interpretation.

Clinical, endocrine, and uterine hemodynamic variables in mares carrying stallion- and donkey-derived pregnancies at Day 49 of gestation, and total number of supplementary corpora lutea (SCL) up to Day 98. Data are presented as mean ± standard deviation or median (interquartile range, Q1–Q3), according to data distribution. Comparisons between groups were performed using Student’s *t*-test or the Mann–Whitney test, as appropriate. The number of animals (*n*) varied across variables because detectable equine chorionic gonadotropin (eCG) concentrations and supplementary corpora lutea (SCL) were observed only in a subset of mares carrying donkey-derived pregnancies. Consequently, analyses related to luteal function and vascularization were restricted to mares with detectable SCL.

### Hormonal and luteal dynamics during early gestation

Longitudinal analysis revealed distinct temporal patterns between stallion- and donkey-derived pregnancies (Table 2).

**Table 2:** Cumulative number of supplementary corpora lutea (SCL) in mares carrying stallion- and donkey-derived pregnancies during early gestation

Variable	GS (*n* = 6)	GD (*n* = 8)	*P*-value
Cumulative SCL – Day 49	1.0 (1.0–2.0)	0 (0–0.5)	0.008
Cumulative SCL – Day 63	1.5 (1.0–2.0)	0 (0–0.5)	0.006
Cumulative SCL – Day 84	2.0 (1.5–3.0)	0 (0–0.75)	0.005
Cumulative SCL – Day 98	2.5 (2.0–3.3)	0.5 (0–1.8)	0.005

Supplementary corpora lutea were detected in all mares carrying stallion-derived pregnancies (GS, 6/6; 100%). In contrast, four of the eight mares carrying donkey-derived pregnancies (GD, 4/8; 50%) did not develop detectable supplementary corpora lutea at any evaluated time point, whereas the remaining four mares (4/8; 50%) developed SCL, although in consistently lower cumulative numbers than those observed in the GS group. Accordingly, the cumulative number of SCL increased progressively in the GS group, whereas consistently lower cumulative values were observed in the GD group across Days 49, 63, 84, and 98 (Table 2). Significant differences between groups were detected at all evaluated time points: Day 49 (*P* = 0.008), Day 63 (*P* = 0.006), Day 84 (*P* = 0.005), and Day 98 (*P* = 0.005).

Hemorrhagic anovulatory follicles (HAF) were identified in two of the six mares carrying stallion-derived pregnancies (2/6; 33.3%) and in one of the eight mares carrying donkey-derived pregnancies (1/8; 12.5%). As described in the Materials and Methods, these structures were excluded from the cumulative SCL counts.

Longitudinal changes in the cumulative number of supplementary corpora lutea (SCL) detected by transrectal ultrasonography on Days 49, 63, 84, and 98 of gestation in mares carrying stallion-derived (GS, *n* = 6) or donkey-derived (GD, *n* = 8) pregnancies. Data are presented as median (interquartile range [IQR]). Comparisons between groups at each gestational time point were performed using the Mann–Whitney U test. Four mares in the GD group did not develop detectable supplementary corpora lutea throughout the experimental period; therefore, the cumulative SCL values presented for the GD group reflect the entire study population (*n* = 8), including these mares. GS = mares carrying stallion-derived pregnancies; GD = mares carrying donkey-derived pregnancies; SCL = supplementary corpora lutea; IQR = interquartile range.

In mares carrying stallion-derived pregnancies, significant changes over time were observed only for luteal vascularization, in both subjective and objective (PIX) assessments (*P* < 0.001). In contrast, mares carrying donkey-derived pregnancies exhibited significant temporal variation in multiple parameters, including progesterone (P4; *P* = 0.002), equine chorionic gonadotropin (eCG; *P* < 0.001), subjective and objective luteal vascularization (*P* < 0.001), pulsatility index (PI) of the non-pregnant uterine horn (*P* = 0.038), and resistivity index (RI) of the same horn (*P* = 0.003; Supplementary Table 1).

Pairwise comparisons revealed group-specific temporal patterns. In stallion-derived pregnancies, subjective and objective luteal vascularization increased between Days 49 and 84 (*P* = 0.030 and *P* = 0.025, respectively) and between Days 49 and 98 (*P* = 0.048 and *P* = 0.010, respectively), with no significant differences among the subsequent time points. In donkey-derived pregnancies, P4 and eCG concentrations decreased between Days 49 and 98 (*P* = 0.029 and *P* = 0.037, respectively) and between Days 63 and 98 (*P* = 0.015 and *P* = 0.016, respectively). Luteal vascularization increased over time in this group in both subjective and objective assessments (*P* < 0.001 for the overall time effect), although not all pairwise comparisons between consecutive time points reached statistical significance.

Regarding uterine hemodynamics, mares carrying donkey-derived pregnancies showed significant temporal changes in PI and RI of the non-pregnant uterine horn. PI decreased between Days 49 and 63 (*P* = 0.024), whereas RI decreased between Days 49 and 98 (*P* = 0.026).

Overall, stallion-derived pregnancies exhibited higher P4 (*P* = 0.001), eCG (*P* = 0.001), and uterine Doppler indices (PI and RI in both uterine horns; *P* < 0.001) across the experimental period. Significant group × time interactions were observed for P4 (*P* = 0.001), objective luteal vascularization (PIX; *P* = 0.036), and RI of the non-pregnant uterine horn (*P* = 0.013; Supplementary Table 2).

### Associations between endocrine and uterine hemodynamic parameters

Spearman correlation analysis identified specific associations between relative changes in endocrine and uterine hemodynamic parameters (Supplementary Table S3).

A strong positive correlation was observed between the relative change in P4 and the relative change in PI of the pregnant uterine horn (*r* = 0.846, *p* = 0.001; *n* = 14). In addition, P4 showed a moderate positive correlation with the relative change in RI of the non-pregnant uterine horn (*r* = 0.596, *p* = 0.024; *n* = 14).

Similarly, the relative change in eCG was positively correlated with the relative change in PI of the pregnant uterine horn (*r* = 0.733, *P* = 0.015; *n* = 10). No other statistically significant associations were detected in the total sample (Table 3).

**Table 3:** Significant Spearman’s rank correlations between relative changes in endocrine parameters and uterine hemodynamic indices in the total sample

Variable 1	Variable 2	*r*	*p*-value	*n*
ΔP4 (%)	**ΔPI (pregnant uterine horn)**	0.846	0.001	14
ΔP4 (%)	**ΔRI (non-pregnant uterine horn)**	0.596	0.024	14
ΔeCG (%)	**ΔPI (pregnant uterine horn)**	0.733	0.015	10

Data are presented as Spearman’s rank correlation coefficients (*r*), exact *P* values, and number of observations (n). Only statistically significant associations identified in the total sample are shown. Relative changes (Δ%) were calculated as [(Day 98 − Day 49)/Day 49] × 100. Statistical significance was set at *P* < 0.05. Correlation analyses were considered exploratory because of the limited sample size.

## Discussion

The present study evaluated endocrine, luteal, and uterine hemodynamic patterns in mares carrying intra- and interspecific pregnancies, demonstrating that conceptus origin is associated with distinct physiological profiles during early gestation.

Consistent with previous reports, mares carrying stallion-derived pregnancies exhibited higher circulating concentrations of progesterone (P4) and equine chorionic gonadotropin (eCG), as well as a greater number of supplementary corpora lutea (SCL). These differences are well established and are primarily attributed to the genotype of the conceptus and its interaction with the maternal endometrium, particularly through trophoblastic signaling and endometrial cup development, rather than a direct systemic effect of the sire (Allen and Stewart 2001; Boeta and Zarco 2005, 2012).

In contrast, donkey-derived pregnancies were characterized by reduced eCG secretion, lower SCL formation, and a progressive decline in circulating P4, consistent with endocrine patterns previously described in interspecific gestations. Moreover, the persistently lower cumulative number of supplementary corpora lutea observed throughout the experimental period further supports the reduced luteal support associated with interspecific pregnancies. The progressive endocrine decline observed in donkey-derived pregnancies is consistent with previous studies demonstrating impaired endometrial cup development and reduced trophoblastic survival in interspecific equine gestations. Allen et al. (1993) and Enders et al. (1996) demonstrated that conceptus genotype influences trophoblastic invasion and endometrial cup formation, resulting in reduced eCG secretion. This phenomenon has been associated with increased maternal immune recognition of fetal tissues and earlier regression of endometrial cup function, ultimately limiting supplementary luteal support (Canisso et al. 2019).

The lower circulating progesterone concentrations observed in mares carrying donkey-derived pregnancies are likely explained, at least in part, by the reduced formation of supplementary corpora lutea, resulting in a lower cumulative luteal tissue available for progesterone production. Interestingly, despite these endocrine differences, luteal vascularization did not differ between groups, suggesting that the lower progesterone concentrations were more closely associated with the reduced number of supplementary corpora lutea than with differences in vascularization of individual luteal structures.

In addition to endocrine differences, the present study identified distinct uterine hemodynamic patterns between groups. The present evaluation was performed in the dorsal branches of the uterine arteries rather than in the middle uterine artery, following a standardized protocol previously adopted by our research group. This approach allows independent assessment of blood flow associated with each uterine horn and facilitates comparisons between pregnant and non-pregnant horns, which is particularly relevant in studies investigating conceptus-related uterine adaptations (Bollwein et al. 2004; Lemos et al. 2017). Mares carrying stallion-derived pregnancies consistently exhibited higher uterine artery Doppler indices (PI and RI), whereas donkey-derived pregnancies showed lower values and a progressive decline over time, particularly in the non-pregnant uterine horn. These findings indicate that conceptus origin is associated with different patterns of vascular adaptation during early pregnancy and extend current knowledge by integrating endocrine and Doppler-derived parameters within the same experimental framework.

From a physiological perspective, uterine artery Doppler indices are inversely related to tissue perfusion, with lower PI and RI values reflecting reduced vascular resistance and increased blood flow (Ginther and Utt 2004; Bollwein et al. 2016). In equine pregnancy, both indices are expected to decrease progressively as gestation advances, reflecting the transition from a relatively high-resistance vascular system during early pregnancy to a low-resistance, high-flow circulation that supports increasing fetal and placental metabolic demands (Bollwein et al. 2004; Ousey et al. 2012; Oliveira et al. 2026). Accordingly, the progressive decline observed in donkey-derived pregnancies may represent a dynamic adjustment in uterine perfusion rather than a pathological condition. However, as direct measurements of uterine blood flow were not performed, this interpretation should be considered descriptive rather than mechanistic.

Because uterine perfusion directly influences placental nutrient and oxygen delivery, differences in maternal vascular adaptation may also have implications for fetal growth and neonatal vigor. Although neonatal outcomes were not evaluated in the present study, future investigations should determine whether the endocrine and hemodynamic patterns identified here are associated with differences in birth weight, neonatal adaptation, or postnatal development.

Previous studies have shown that uterine blood flow progressively increases throughout gestation in mares, reflecting the growing metabolic demands of the developing conceptus (Bollwein et al. 2004; Ousey et al. 2012). More recently, Oliveira et al. (2026) reported lower uterine artery Doppler indices in mares carrying donkey-derived pregnancies compared with horse pregnancies, supporting the concept that conceptus origin influences maternal vascular adaptation. The present findings are consistent with these observations and further demonstrate that these hemodynamic differences occur concurrently with distinct endocrine profiles.

The association between endocrine profiles and uterine hemodynamics observed in this study suggests a coordinated interaction between hormonal support and vascular adaptation during early gestation. Nevertheless, this relationship should be interpreted with caution, as the study design does not allow causal inference. Importantly, progesterone production in equine pregnancy is not exclusively dependent on supplementary corpora lutea, as both the primary corpus luteum and, subsequently, the fetoplacental unit contribute to progestagen synthesis (Allen and Stewart 2001). Therefore, the reduced number of SCL observed in donkey-derived pregnancies should not be interpreted as evidence of intrinsic luteal dysfunction. Although these observations suggest a physiological association between endocrine support and uterine vascular adaptation, the present data do not allow us to determine whether one mechanism directly influences the other.

Although causal relationships cannot be established from the present data, similar associations between luteal function, circulating progesterone concentrations, and reproductive blood flow have been reported in other species. Studies in sheep, goats, and cattle have demonstrated positive relationships between luteal vascularization, progesterone secretion, and reproductive tract perfusion (Figueira et al. 2015; Balaro et al. 2017; Pugliesi et al. 2017). Together, these findings support the concept that endocrine and vascular adaptations are closely interconnected components of reproductive physiology.

Luteal vascularization increased over time in both groups, consistent with the established relationship between luteal perfusion and steroidogenic activity. Despite differences in temporal patterns, no differences were observed between groups in absolute vascularization values, indicating that local luteal structural adaptation is preserved regardless of conceptus origin. This finding suggests that luteal vascular dynamics remain functionally adequate even under distinct endocrine environments. This apparent dissociation suggests that factors other than local luteal perfusion contributed to the endocrine differences observed between groups. In addition, the distinct uterine hemodynamic profile identified in donkey-derived pregnancies may reflect differences in maternal vascular adaptation associated with conceptus origin, although its relationship with circulating progesterone concentrations remains unclear. Further studies are warranted to clarify these mechanisms.

Despite the endocrine and hemodynamic differences identified, all pregnancies included in the study were maintained to term. This observation reinforces that equine gestation can be sustained across a range of physiological conditions and supports previous evidence indicating that supplementary corpora lutea are not strictly required for pregnancy maintenance, but may act as a supportive mechanism under specific conditions (Boeta and Zarco 2012).

Notably, a subset of mares carrying donkey-derived pregnancies exhibited persistently undetectable eCG concentrations and absence of supplementary corpora lutea throughout the experimental period, yet maintained pregnancy to term. The persistently undetectable eCG concentrations observed in four of eight donkey-derived pregnancies may indicate that endometrial cups were present but markedly reduced in size or functional activity, resulting in circulating eCG concentrations below the assay detection limit. These animals were excluded from quantitative analyses involving eCG concentrations, supplementary corpora lutea, and luteal vascularization because values below the assay detection limit could not be reliably incorporated into statistical comparisons and would potentially introduce analytical bias. However, they were retained in all other analyses and considered in the descriptive interpretation of the results. Biologically, this subgroup is particularly interesting because it demonstrates that pregnancy maintenance can occur despite the apparent absence of eCG-induced luteal support. These observations reinforce the concept that, although eCG contributes to supplementary luteal formation and progesterone production, it is not strictly required for gestational success. Compensatory mechanisms involving sustained primary corpus luteum function and/or earlier reliance on fetoplacental progestagen production may account for this outcome (Allen et al. 1993; Enders et al. 1996), highlighting the remarkable physiological adaptability of equine pregnancy.

The main contribution of the present study lies in the integrated evaluation of endocrine, luteal, and uterine hemodynamic parameters. While endocrine differences in interspecific pregnancies have been extensively described, their association with uterine vascular patterns has been less explored. Thus, the present findings provide additional descriptive insight into maternal adaptations associated with conceptus origin, without implying novel mechanistic pathways.

This study presents important limitations that should be acknowledged. The small sample size limits statistical power and restricts broader generalization of the findings. The use of a limited number of sires may introduce individual variability that cannot be fully separated from conceptus-related effects. Group allocation was not formally randomized, as it followed a pre-established breeding program of the experimental farm; although no significant baseline differences were identified between groups in age or body condition score, the influence of unmeasured individual factors cannot be fully excluded.

Mare age should also be considered as a potential source of biological variation, since age-related differences in uterine hemodynamics and reproductive performance have been reported in pregnant mares (Ousey et al. 2012; Carluccio et al. 2020). Although no significant age differences were observed between groups in the present study, the relatively small sample size limits the ability to completely exclude residual age-related effects.

The weekly ultrasonographic monitoring schedule, while sufficient for continuous tracking of ovarian dynamics throughout the experimental period, does not provide the same diagnostic precision as daily monitoring for definitive characterization of supplementary luteal structures in all cases. Although the combined use of B-mode and power Doppler ultrasonography provided complementary morphological and functional criteria for differentiation between true SCL and hemorrhagic anovulatory follicles, the possibility of occasional misclassification cannot be entirely excluded.

Finally, neonatal outcomes were not evaluated, preventing assessment of the functional consequences of the endocrine and hemodynamic differences identified. Future studies with larger sample sizes, formal randomization, daily ovarian monitoring, and evaluation of neonatal outcomes are warranted.

In conclusion, conceptus origin was associated with distinct endocrine, luteal, and uterine hemodynamic profiles during early equine pregnancy. Stallion-derived pregnancies exhibited higher P4 and eCG concentrations, greater SCL formation, and higher uterine Doppler indices, whereas donkey-derived pregnancies were characterized by reduced hormonal support and lower vascular resistance indices over time. These findings reflect differences in maternal physiological adaptation associated with conceptus genotype rather than a direct sire effect. Despite these differences, all pregnancies were maintained to term, indicating that gestational success may occur under variable endocrine and hemodynamic conditions. These observations are restricted to the gestational period characterized by maximal endometrial cup activity and eCG secretion and should not be extrapolated to other stages of equine pregnancy without further investigation. The integration of hormonal, luteal, and Doppler parameters provides a more comprehensive characterization of maternal adaptations associated with conceptus origin and may contribute to future monitoring strategies in reproductive programs.

## Supplementary Information

Below is the link to the electronic supplementary material.

Supplementary Material 1

## Data Availability

The datasets generated and/or analyzed during the current study are available from the corresponding author on reasonable request.
