# Supplementary material for: Endocrine and uterine hemodynamic differences between intra- and interspecific equine pregnancies during early gestation
Source: Vet Res Commun. 2026 Aug 25;50(6):527. doi: 10.1007/s11259-026-11472-1 (PMC13506588; doi:10.1007/s11259-026-11472-1)
Supplement: Supplementary file 1 [file 11259_2026_11472_MOESM1_ESM.docx]

**Supplementary Table 1.** Longitudinal descriptive analysis of endocrine, luteal, and uterine hemodynamic parameters in mares carrying stallion- and donkey-derived pregnancies from Day 49 to Day 98 of gestation.

| **Parameter** | **Groupo** | **n** |  | **D1 (Day 49)** | | | **D2 (Day 63)** | | | **D3 (Day 84)** | | | **D4 (Day 98)** | | | **NOVAᵃ (time)** |
| --- | --- | --- | --- | --- | --- | --- | --- | --- | --- | --- | --- | --- | --- | --- | --- | --- |
|  |  |  |  |  |  |  |  |  |  |  |  |  |  |  |  |  |
|  |  |  |  |  |  |  |  |  |  |  |  |  |  |  |  |  |
| **P4; ng/mL** | Stallion | 6 |  | 25,6 | ± | 5,0 | 40,3 | ± | 9,8 | 40,7 | ± | 9,3 | 40,9 | ± | 9,0 | 0,072 |
|  | Donkey | 8 |  | 14,3 | ± | 1,8 | 13,6 | ± | 1,8 | 12,1 | ± | 1,7 | 10,8 | ± | 1,3 | **0,002** |
|  |  |  |  |  |  |  |  |  |  |  |  |  |  |  |  |  |
| **eCG; IU/mL** | Stallion | 6 |  | 88,7 | ± | 3,2 | 87,5 | ± | 5,2 | 78,7 | ± | 7,1 | 64,5 | ± | 10,3 | 0,083 |
|  | Donkey | 4 |  | 56,0 | ± | 4,9 | 57,5 | ± | 3,2 | 40,0 | ± | 3,4 | 29,5 | ± | 2,9 | **0,0001** |
|  |  |  |  |  |  |  |  |  |  |  |  |  |  |  |  |  |
| **Subjective luteal vascularization (score)** | Stallion | 6 |  | 16,7 | ± | 4,8 | 33,3 | ± | 6,7 | 48,3 | ± | 6,0 | 53,3 | ± | 4,2 | **0,0001** |
|  | Donkey | 4 |  | 18,8 | ± | 4,3 | 42,5 | ± | 4,8 | 62,5 | ± | 4,8 | 73,8 | ± | 2,4 | **< 0,0001** |
|  |  |  |  |  |  |  |  |  |  |  |  |  |  |  |  |  |
| **Objective luteal vascularization (PIX)** | Stallion | 6 |  | 5015 | ± | 1261,7 | 9212 | ± | 1952 | 12584 | ± | 2035 | 14159 | ± | 2070 | **< 0,0001** |
|  | Donkey | 4 |  | 2890 | ± | 146,8 | 11131 | ± | 828 | 14042 | ± | 2039 | 21202 | ± | 553 | **< 0,0001** |
|  |  |  |  |  |  |  |  |  |  |  |  |  |  |  |  |  |
| **Pulsatility index (PI) of the pregnant uterine horn** | Stallion | 6 |  | 2,16 | ± | 0,07 | 2,27 | ± | 0,08 | 2,43 | ± | 0,07 | 2,51 | ± | 0,12 | 0,063 |
|  | Donkey | 8 |  | 1,35 | ± | 0,03 | 1,31 | ± | 0,02 | 1,31 | ± | 0,03 | 1,31 | ± | 0,04 | 0,48 |
|  |  |  |  |  |  |  |  |  |  |  |  |  |  |  |  |  |
| **Pulsatility index (PI) of the non-pregnant uterine horn** | Stallion | 6 |  | 1,93 | ± | 0,04 | 1,92 | ± | 0,05 | 1,79 | ± | 0,07 | 1,94 | ± | 0,08 | 0,11 |
|  | Donkey | 8 |  | 1,17 | ± | 0,03 | 1,11 | ± | 0,02 | 1,14 | ± | 0,03 | 1,15 | ± | 0,03 | **0,038** |
|  |  |  |  |  |  |  |  |  |  |  |  |  |  |  |  |  |
| **Resistivity index (RI) of the pregnant uterine horn** | Stallion | 6 |  | 0,82 | ± | 0,01 | 0,84 | ± | 0,02 | 0,87 | ± | 0,02 | 0,84 | ± | 0,02 | 0,27 |
|  | Donkey | 8 |  | 0,65 | ± | 0,03 | 0,67 | ± | 0,03 | 0,65 | ± | 0,02 | 0,60 | ± | 0,04 | 0,066 |
|  |  |  |  |  |  |  |  |  |  |  |  |  |  |  |  |  |
| **Resistivity index (RI) of the non-pregnant uterine horn** | Stallion | 6 |  | 0,79 | ± | 0,03 | 0,86 | ± | 0,03 | 0,78 | ± | 0,02 | 0,84 | ± | 0,02 | 0,13 |
|  | Donkey | 8 |  | 0,42 | ± | 0,05 | 0,40 | ± | 0,04 | 0,35 | ± | 0,02 | 0,35 | ± | 0,03 | **0,003** |

Data are presented as mean ± standard error (SE). Repeated measures analysis of variance (ANOVA) was used to evaluate temporal changes within each group (time effect). D1 = Day 49, D2 = Day 63, D3 = Day 84, and D4 = Day 98 of gestation. The number of animals (n) varied across variables due to the absence of supplementary corpora lutea in some mares, particularly in the donkey-derived group. Values in bold indicate statistically significant differences over time within each group (*p* < 0.05).

**Supplementary Table 2.** Pairwise comparisons and mixed-model repeated measures ANOVA evaluating the effects of time, group, and group × time interaction on endocrine, luteal, and uterine hemodynamic parameters.

| **Parameter** | **Group** | **n** |  |  | **Multiple comparisonsᵇ** | |  |  |  |  |  | **ANOVA with factorsᶜ** | |
| --- | --- | --- | --- | --- | --- | --- | --- | --- | --- | --- | --- | --- | --- |
|  |  |  |  |  | **D1**  **vs**  **D2** | **D1**  **Vs**  **D3** | **D1**  **vs**  **D4** | **D2**  **vs**  **D3** | **D2**  **vs**  **D4** | **D3 vs D4** |  | **group** | **interaction** |
|  |  |  |  |  |  |  |  |  |  |  |  |  |  |
| **P4; ng/mL** | Stallion | 6 |  |  | 0,69 | 0,55 | 0,58 | 0,97 | 0,99 | 0,99 |  | **0,001** | **0,001** |
|  | Donkey | 8 |  |  | 0,96 | 0,48 | **0,029↓** | 0,50 | **0,015↓** | 0,42 |  |  |  |
|  |  |  |  |  |  |  |  |  |  |  |  |  |  |
| **eCG; IU/mL** | Stallion | 6 |  |  | 0,99 | 0,79 | 0,48 | 0,39 | 0,29 | 0,51 |  | **0,001** | 0,26 |
| **eCG** | Donkey | 4 |  |  | 0,96 | 0,42 | **0,037↓** | 0,18 | **0,016↓** | 0,15 |  |  |  |
|  |  |  |  |  |  |  |  |  |  |  |  |  |  |
| **Subjective luteal vascularization (score)** | Stallion | 6 |  |  | 0,07 | **0,030↑** | **0,048↑** | 0,09 | 0,31 | 0,93 |  | 0,21 | 0,99 |
| **Avalição subjetiva** | Donkey | 4 |  |  | **0,037↑** | **0,024↑** | **0,037↑** | **0,014↑** | **0,040↑** | 0,44 |  |  |  |
|  |  |  |  |  |  |  |  |  |  |  |  |  |  |
| **Objective luteal vascularization (PIX)** | Stallion | 6 |  |  | 0,07 | **0,025↑** | **0,010↑** | 0,06 | 0,35 | 0,98 |  | 0,55 | **0,036** |
| **Avaliação objetiva PIX** | Donkey | 4 |  |  | **< 0,001↑** | **0,004↑** | **< 0,001↑** | 0,43 | **0,013↑** | 0,26 |  |  |  |
|  |  |  |  |  |  |  |  |  |  |  |  |  |  |
| **Pulsatility index (PI) of the pregnant uterine horn** | Stallion | 6 |  |  | 0,95 | 0,47 | 0,33 | 0,67 | 0,63 | 0,99 |  | **< 0,0001** | 0,10 |
| **PI Corno Gestante** | Donkey | 8 |  |  | 0,66 | 0,88 | 0,85 | 0,99 | 0,99 | 0,99 |  |  |  |
|  |  |  |  |  |  |  |  |  |  |  |  |  |  |
| **Pulsatility index (PI) of the non-pregnant uterine horn** | Stallion | 6 |  |  | 0,99 | 0,70 | 0,99 | 0,13 | 0,99 | 0,10 |  | **< 0,0001** | 0,081 |
| **PI Corno não-Gestante** | Donkey | 8 |  |  | **0,024↓** | 0,08 | 0,85 | 0,82 | 0,60 | 0,99 |  |  |  |
|  |  |  |  |  |  |  |  |  |  |  |  |  |  |
| **Resistivity index (RI) of the pregnant uterine horn** | Stallion | 6 |  |  | 0,94 | 0,31 | 0,97 | 0,69 | 0,99 | 0,88 |  | **< 0,0001** | 0,11 |
| **RI Corno Gestante** | Donkey | 8 |  |  | 0,26 | 0,99 | 0,50 | 0,93 | 0,12 | 0,50 |  |  |  |
|  |  |  |  |  |  |  |  |  |  |  |  |  |  |
| **Resistivity index (RI) of the non-pregnant uterine horn** | Stallion | 6 |  |  | 0,52 | 0,99 | 0,92 | 0,22 | 0,99 | 0,49 |  | **< 0,0001** | **0,013** |
| **RI Corno não-Gestante** | Donkey | 8 |  |  | 0,51 | 0,16 | **0,026↓** | 0,44 | 0,17 | 0,99 |  |  |  |

Sidak-adjusted multiple comparisons were performed to identify significant differences between time points within each group. Mixed-model repeated measures ANOVA was used to assess the effects of group, time, and group × time interaction. Data are presented as mean ± standard error (SE). D1 = Day 49, D2 = Day 63, D3 = Day 84, and D4 = Day 98 of gestation. Data were log-transformed using the natural logarithm (ln) when necessary to meet model assumptions. Statistical significance was set at *p* < 0.05. Values in bold indicate statistically significant differences. Arrows (↑, ↓) denote the direction of significant changes between time points within each group, where ↑ indicates an increase and ↓ indicates a decrease relative to the preceding time point.

**Table S3.** Spearman correlation coefficients between relative changes (%) in endocrine parameters (ΔP4 and ΔeCG) and relative changes in uterine hemodynamic indices (ΔPI and ΔRI) in pregnant and non-pregnant uterine horns, evaluated in the total sample and stratified by group (stallion- and donkey-derived pregnancies).

Data are presented as Spearman correlation coefficient (r), p-value, and number of observations (n). Relative change (%) was calculated as: [(Day 98 − Day 49) / Day 49] × 100. Positive values indicate an increase over time, whereas negative values indicate a decrease. Correlations are shown for the total sample and separately for stallion- and donkey-derived pregnancies. Statistical significance was set at p < 0.05.
